# “Test Your Spirituality in One Minute or Less” Structural Validity of the Multidimensional Inventory for Religious/Spiritual Well-Being Short Version (MI-RSWB 12)

**DOI:** 10.3389/fpsyg.2021.597565

**Published:** 2021-02-02

**Authors:** Jürgen Fuchshuber, Human F. Unterrainer

**Affiliations:** ^1^Center for Integrative Addiction Research (CIAR), Grüner Kreis Society, Vienna, Austria; ^2^University Clinic for Psychiatry and Psychotherapeutic Medicine, Medical University Graz, Graz, Austria; ^3^Department of Religious Studies, University of Vienna, Vienna, Austria

**Keywords:** psychological well-being, scale validation, spirituality structural equation modeling, test development, spirituality

## Abstract

**Background:** The Multidimensional Inventory for Religious/Spiritual Well-Being (MI-RSWB 48) was developed in order to address a religious/spiritual dimension as being an important part of psychological well-being. In the meantime, the instrument has been successfully applied in numerous studies. Subsequently, a short version, the MI-RSWB 12 was constructed, especially for the use in clinical assessment. Here it is intended to contribute to the further development of the MI-RSWB 12 by investigating its structural validity through structural equation modeling.

**Materials and Methods:** A total sample of 1,097 German-speaking adults (744 females; 67.8%; Age range: 18–69 years) from the normal population filled in the MI-RSWB 12 via an online-survey. In line with theoretical assumptions 5 different factor structure models for the MI-RSWB 12 were tested: (1) a single-factor model, (2) a model with four correlated RSWB dimensions, (3) a single higher-order model with four lower order factors, (4) a two higher-order model with four lower order factors, (5) a bifactor model, which includes four specific RSWB dimensions.

**Results:** The single-factor model provided the poorest model fit, with no indices falling within the acceptable range. The four-factor, two higher-order factors and the bifactor models showed overall acceptable fit indices. With regard to the Akaike information criterion (AIC), the four-factor model demonstrated superiority compared to both the two higher-order factor model and the bifactor model, which in turn showed did not differ from each other.

**Conclusion:** Four different MI-RSWB 12 sub-scales should be calculated in future studies, while a general factor and two higher order factors are statistically valid as well. Further applications of the MI-RSWB 12, especially in the clinical patient groups, are encouraged.

## Introduction

In the past two decades in particular, there has been a remarkable increase in interest coming from psychology, medicine as well as the whole field of neurosciences in topics such as religiosity and spirituality as being related to various parameters of mental health, psychological well-being, and coping with illness (cf. Rosmarin et al., 2020). This comes rather surprisingly, as originally the whole field of natural sciences viewed religion rather critically. Accordingly, Sigmund Freud rated religion as a kind of obsessive-compulsive neurosis (Freud, 1907) as well as an illusion, which will dissolve in the future (Freud, 1927). But not only Freud, as the founder of psychoanalysis, also one of the earliest representatives of behavioral theories, Ivan Pavlov, could find little positive in religion, as he judged religion as a kind reassurance for “nervenschwache Menschen” [neurasthenic people] (cf. Windholz, 1986). In contrast, Jung (1962/1937), the founder of Analytical Psychology, took a completely contrary position and saw religion and spirituality as an integral part of the human psyche (cf. Palmer, 2003; see also Kernberg, 2000 or Black, 2006 for an overview of contemporary psychoanalytic views on religion and spirituality). In correspondence to the positive Jungian view of religion there has been a benevolent appraisal of religion and spirituality throughout the second half of the 20th century, especially in the tradition of humanistic psychology. Correspondingly, one of the key figures of the humanistic approach, Abraham Maslow placed the need for transcendence (or the transpersonal realm in Maslow's words) at the top of his pyramid of needs (1964).

One of the main concerns for the field of psychology of religion has always been to precisely define its own research subject (Pargament, 1999). Both areas of religiosity and spirituality partly overlap in content as they concern the transcendent area of perception, whereby religiosity can be perceived more as a belief system tied to institutions and traditions. In the case of spirituality, the connection to the transcendent realm of perception refers more to the personal relationship of the human individual to God or a higher power of any kind, regardless of a particular belief system (Unterrainer et al., 2014). This lack of a clear definition also affects the psychometric assessment of religiosity and/or spirituality, as various scales and measurement models have been developed based on various theoretical backgrounds (primarily in the Anglo-American area) since the end of the 19th century (Hill and Hood, 1999). Accordingly, Hill and Edwards (2013) provide a brief overview of reliable and valid measures of various aspects of religiousness and spirituality. Here the measures are classified by several categories that fall under two general headings: substantive measures and functional measures. In addition, they put a special focus on cultural sensitivity, as most of the instruments are so far developed within a Judeo-Christian context. Furthermore, Kapuscinski and Masters (2010) critically review the scale development practices for 24 measures of spirituality pertaining to various aspects of conceptualization and psychometric properties. In line with their findings they raise theological as well as theoretical concerns, which should inform future development and validation of spirituality measures.

The concept of Religious/Spiritual Well-Being (RSWB) was developed based the original concept of the Spiritual Well-Being (SWB) scale (Ellison, 1983; Bufford et al., 1991; see also Moberg, 1971 for the original concept of spiritual well-being). This is where we meet for the first time a separation between a transcendent space of perception (which determines the amount of “religious well-being”—RWB) and an immanent space of perception (which determines the amount of “existential well-being”—EWB). The total SWB score results by summing up both sub-scales for EWB and RWB.

In this sense, the Multidimensional Inventory of Religious/Spiritual Well-Being (MI-RSWB 48) may be considered as a multidimensional alternative to the original SWB scale. Therefore, the MI-RSWB 48 was developed in order to meet the unanimous demand for a multidimensional assessment of religiosity and spirituality (Fetzer Institute/National Institute on Aging Working Group, 1999; Unterrainer et al., 2010b). Furthermore, the theoretical background of the MI-RSWB was elaborated based on an interdisciplinary discourse of all professional groups working at the Medical University of Graz/Austria. Within this scientific study group one of the main questions was how to address religious/spiritual issues in patient treatment most adequately. Furthermore, a global definition for Religious/Spiritual Well-Being (RSWB) can be given as “the ability to experience and integrate meaning and purpose in existence through a connectedness with self, others or a power greater than oneself.” (Unterrainer et al., 2011, p. 117).

Notably, there is no strict separation between immanent space and transcendent space of perception in other multidimensional inventories for the assessment of religiosity and spirituality, as for instance the Brief Multidimensional Measure of Religiousness/Spirituality (BMMRS; Fetzer Institute & National Institute on Aging Working Group 1999) differentiates between several religious (e.g., private religious practices) and spiritual dimensions (e.g., meaning), however does not consider a strict differentiation between an immanent and a transcendent area of perception (see also Johnstone et al., 2012 for further discussion) although, for example, the dimension of forgiveness is taken into account in both procedures.

In its original version, the MI-RSWB 48 comprises six dimensions, three dimensions for the immanent area of perception and three dimensions for the transcendent area of perception. All six dimensions can be summed up to a total RSWB total-score. Furthermore, marker items are given as examples in order to illustrate the meaning of the different dimensions. General Religiosity: “My faith gives me a feeling of security”; Connectedness: “I have experienced the feeling of being absorbed into something greater”; Forgiveness: “There are things which I cannot forgive” (coded reverse); Experiences of Sense and Meaning: “I have experienced true (authentic) feelings”; Hope Immanent: “I view the future with optimism”; Hope Transcendent: “I often think about the fact that I will have to leave behind my loved ones.” (coded reverse). The MI-RSWB 48 scale was used in a large number of studies in clinical as well as community samples (see e.g., Unterrainer et al., 2014 or more recently Unterrainer, 2021 for an extensive overview). Subsequently, norm values for the entire Austrian population could be presented (Unterrainer and Fink, 2013). Furthermore, the scale could also translated and validated into several different languages successfully (Unterrainer, 2021; for an recent overview). Thereby, the MI-RSWB mostly displayed appealing psychometric properties as well as robust factor structure (also in different language versions). In summary, the postulated positive association between the RSWB dimensions could be confirmed with various parameters of mental health and more adequate coping with illness, whereby the MI-RSWB sub-dimensions Hope Immanent as well as Forgiveness mostly turned out to be the strongest predictors of subjective well-being (Unterrainer, 2021).

Although the scale was generally well-received in its application, there was some critique regarding the length of the instrument, as the scale was often perceived as too extensive, especially for applications in clinical settings. Therefore, based on a data set representative for the Austrian normal population (Unterrainer and Fink, 2013), a short version of the scale was created with a total amount of 12 items (Unterrainer and Kapfhammer, 2013).

Thereby, in a first step the two psychometrically weakest scales “Experiences of Sense and Meaning” and “Hope Transcendent” were completely deleted from the inventory. As already reported by Unterrainer et al. (2010a; 2010b; see also Unterrainer et al., 2014), omitting the “Hope Transcendent” scale for the long version of the scale would have also led to a better model fit for the MI-RSWB 48. However, it was decided at that time, mainly because of content considerations, to keep the “Hope Transcendent” sub-scale within the inventory. Furthermore, the subscale “Experiences of Sense and Meaning” showed to be always the weakest in terms of content, because the psychometrically stronger scales “Hope Immanent” and “Forgiveness” already covered a broad area of “Immanent Well-Being.” In a second step, the items of remaining the four scales were sorted out according to selectivity by means of reliability analysis. So only the three most selective items per scale remain in the inventory. In a third and last step, a final factor analysis and reliability analysis were conducted, whereby convincing psychometric properties of the scale could be confirmed.

The current study adds up to our previous work, which has been conducted by applying the MI-RSWB in German version. Hereby we intend to validate the same measure in a different sample by utilizing an additional and better suited approach.

### Study Aims

It is intended in this study, to further contribute to the development of the MI-RSWB 12 scale by investigating its underlying factor validity more in detail. Thereby five competing models are to be tested by applying Structure Equation Modeling (SEM).

## Materials and Methods

### Sample and Procedure

The sample was recruited through various social networks. Informed consent was acquired before each participant filled in the test form that included demographic questions as well as the standardized questionnaire described below. The data was acquired via the online-survey platform LimeSurvey. Data was analyzed from all participants that were aged at least 18 years, spoke German fluently and filled in all questionnaires. The study was carried out in accordance with the Declaration of Helsinki. Ethical approval was granted by the Ethics Committee of the Medical University of Graz, Austria.

### Psychometric Assessment

*The Multidimensional Inventory for Religious/Spiritual Well-Being short version* (MI-RSWB 12; Unterrainer and Kapfhammer, 2013) is a self-report measure which assesses different dimensions of spiritual and religious well-being. It is the shortened version of the MI-RSWB 48 (Unterrainer et al., 2010b). Therefore, it consists of 12 items, which are rated on a 6-point Likert scale ranging from 1 (“strongly disagree”) to 6 (“strongly agree”). These 12 items can be summarized to four sub-scales subscales (3 items per scale). Taken together, the subscales “General Religiosity” (GR), “Connectedness” (CO), “Forgiveness” (FO), and “Hope” (HO) are assumed to reflect the total score “Religious/Spiritual Well-Being” (RSWB). Furthermore, due to the theoretical assumption of a differentiation between the immanent and the transcendent area of perception it is possible to summarize the dimensions GR and CO to the sub-score “Transcendent Well-being” (TWB) and the FO and HO dimensions to the sub-score “Immanent Well-being” (IWB). Accordingly, TWB and IWB in turn add up to the total RSWB score (Unterrainer, 2021; see also Luckmann, 1990; Yalom, 2020 for a further discussion of the theoretical underpinnings). Reliability was assessed with McDonald's ω which recent results suggest as a superior indicator compared to the frequently used Cronbachs's α (Dunn et al., 2014; Hayes and Coutts, 2020). McDonald's ω for the subscales were generally acceptable and ranged between ω = 0.69 and ω = 0.90. The total RSWB score showed a McDonald's ω = 0.77. Furthermore, both IWB (ω = 0.72) and TWB (ω = 0.82) indicated acceptable internal consistencies (see Table 1). A list of the English as well of German items together with a short manual can be found in the Appendix (see A1 and A2).

**Table 1:** Multidimensional inventory for religious/spiritual well-being short version (MI-RSWB 12): descriptive statistics.

								**Kolmogorov-Smirnov Test**	
**Variable**	**ω**	**M**	**SD**	**Min**	**Max**	**Skewness**	**Kurtosis**	***z***	***p***
1. General Religiosity	0.90	6.78	4.30	3	18	0.94	−0.29	6.80	< 0.001
2. Connectedness	0.69	7.83	4.01	3	18	0.55	−0.59	3.79	< 0.001
3. Hope	0.87	12.75	3.76	3	18	−0.66	−0.10	3.53	< 0.001
4. Forgiveness	0.75	12.50	3.96	3	18	−0.45	−0.76	3.90	< 0.001
5. IWB	0.72	25.25	6.08	6	36	−0.49	−0.21	2.80	< 0.001
6. TWB	0.82	14.61	7.17	6	36	0.66	−0.34	3.80	< 0.001
7. RSWB	0.70	39.87	10.64	12	72	0.17	−0.24	1.24	0.09

*N = 1,097; ω, McDonald's omega; M, Mean; SD, Standard Deviation; IWB, Immanent Well-Being; TWB, Transcendental Well-Being; RSWB, Religious/Spiritual Well-Being*.

### Statistical Analysis and Analysis Strategy

The Confirmatory Factor Analysis (CFA) was conducted with AMOS 26. SPSS 27.0 was used for data management, descriptive statistics and bivariate correlations. Goodness-of-fit was assessed with maximum likelihood (ML) estimation in AMOS. In accordance with Kline (2015), the following global fit-indices were considered as markers for an acceptable model fit: (a) The Comparative Fit Index (*CFI*) > 0.90; (b) Tucker-Lewis Index (*TLI*) > 0.90; (c) the Normed Fit Index (NFI) > 0.90; (d) the square root error of approximation (*RMSEA*) < 0.08 and the upper bound of its 90% confidence interval < 1; and (e) χ^2^/df < 3. The χ^2^ significance test was neglected in this analysis as marker of good model fit, as a non-significant χ^2^ test is rarely obtained with large samples (Jöreskog and Sörbom, 1993; Hooper et al., 2008).

For the comparison of competing models, the Akaike Information Criterion (*AIC*) was used, which rewards models that achieve a high goodness-of-fit and penalizes them if they become overly complex (Kline, 2015). In this context, the model with the smallest AIC value was preferred, with a ΔAIC > 2 indicating significant differences (Cheung and Rensvold, 2002; Jovanović, 2015).

What is more, the most parsimonious model was tested for gender invariance via multigroup analysis conducted in Amos. In order to statistically evaluate the differences across the groups, a test of invariance based on a difference in CFI was performed. For this aim, the sequential constraint approach suggested by Dimitrov (2010) was carried out. In correspondence to this, first, it was tested whether the factor loading pattern is the same across groups (metric invariance) by constraining all factor loadings and comparing this model to the unconstrained baseline model. Second, to examine scalar invariance the model with constrained factor loadings was contrasted by a model with additionally constrained item intercepts. Third, for the assessment of invariance of item uniqueness this model was compared to a model which also constrained residual item variances and covariances. Finally, the last model included constrained factor loadings, item intercepts, factor variances and covariances. A non-significant difference between this model and the second model (constrained factor loadings and item intercepts) suggests structural invariance. In each step a ΔCFI ≥ −0.01 was the criterion by which the null hypothesis that the model was equal across the groups was rejected (Cheung and Rensvold, 2002; Dimitrov, 2010). While there is evidence that ML estimation is relatively robust in terms of using non-normal data (Nevitt and Hancock, 2001), Bollen-Stine bootstrap was used in this study to manage the effects of non-normality in the investigated dataset, as studies have also demonstrated that ML test statistic and ML parameter standard error might be affected in the case of a severe violation of assumption of multivariate normal distribution (Kline, 2015). In correspondence to this, Bollen-Stine bootstrap enables a more realistic estimation of standard errors. In accordance with Nevitt and Hancock (2001), 2,000 bootstrap samples were drawn to assess overall model fit and 250 bootstrap samples to obtain parameter estimates and standard errors.

**Figure 1:**
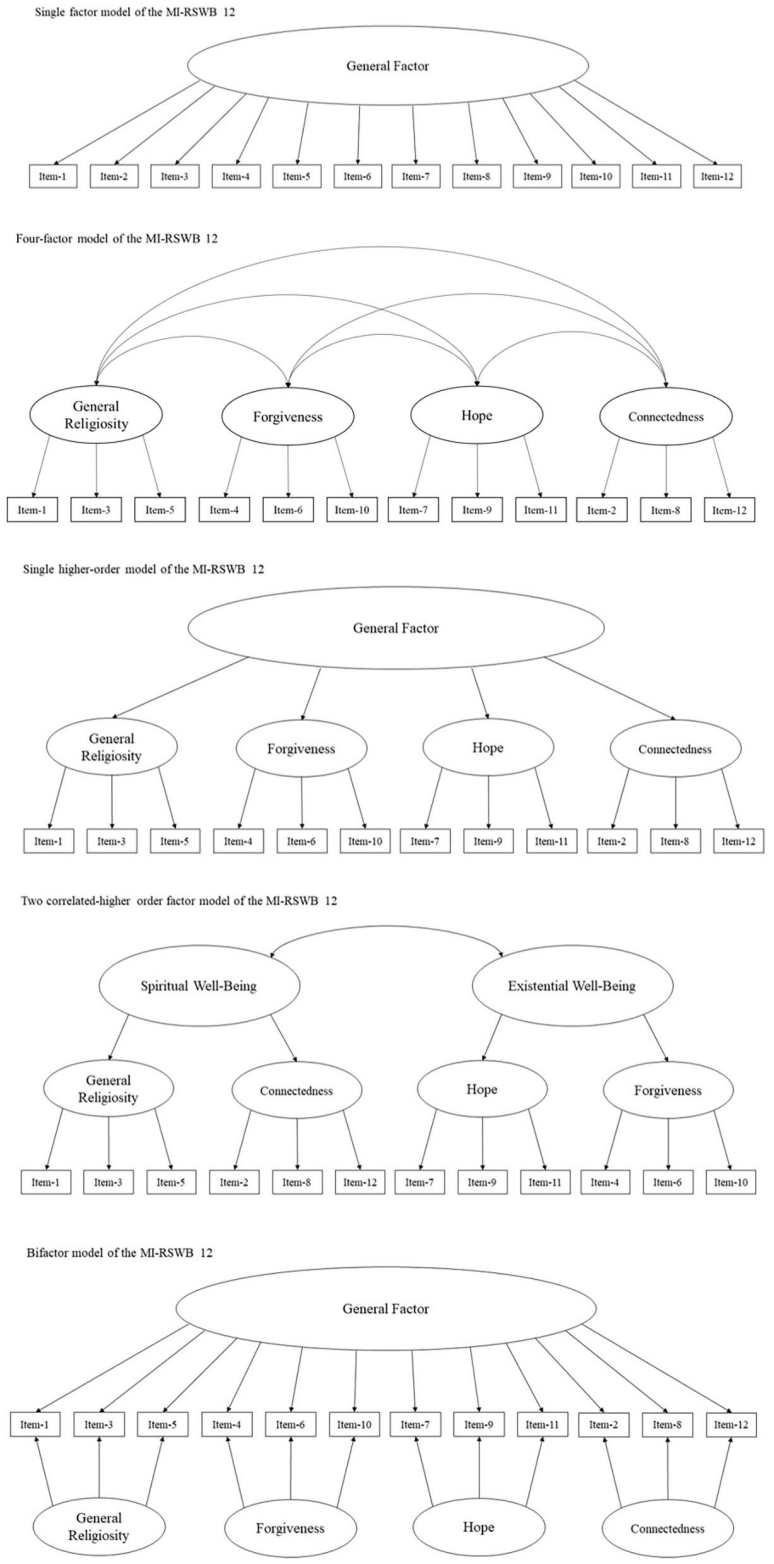
Investigated latent factor models of the MI-RSB 12.

Based on theory and previous research outlined above, five models of the MI-RSWB 12 were tested (see Figure 1): (1) A single factor model which loaded all 12 items onto one underlying factor of RSWB; (2) a model with four correlated dimensions of RSWB; (3) a single higher-order model with four lower order factors and one higher order factor which accounts for the shared variances by the lower order factors. In this model, the items load onto the lower order factors, while the lower order factors load onto the higher order factor; (4) a two higher-order model with four lower order factors and two higher order factor which accounts for the shared variances by two lower order factors each. In this model, the items load onto the lower order factors, while the lower order factors GR and CO load onto the higher order factor TWB and the lower order factors HO and FO load onto the higher order factor IWB; and (5) a bifactor model, including four specific dimensions of RSWB and a general factor. In the bifactor model, each item loads onto both the general factor and the specific factors. To establish model identification one factor loading was fixed to 1 for each factor in every specified model (Byrne, 2004).

## Results

### Sample Characteristics and Descriptive Statistics

The investigated sample consisted of 1,097 German-speaking adults (744 females; 67.8%). The participants ranged in age from 18 to 69 years (*M* = 26.27; *SD* = 8.00). Most participants declared a general qualification for university entrance as their highest educational level (*n* = 514; 46.9%). 396 (36.1%) participants declared a university degree as their highest educational level, 46 (4.2%) a high school degree and, 96 (8.8%) participants stated a completed apprenticeship as their highest educational level. 29 (2.5%) participants stated that they left school without graduation. Most participants stated to be religiously affiliated to Catholicism (635; 57.9%), 358 (32.6%) were without any affiliation, 55 (5%) were protestant, 33 (3%) were members of other Christian religious communities (e.g., orthodox Christianity), while 16 (1.5%) identified themselves with other non-Christian religious communities (e.g., Buddhism). The nationality of most participants was either German (*n* = 354; 32.3%), Austrian (*n* = 659; 60%) or Swiss (*n* = 50; 4.6%), while 50 (4.6%) stated other nationalities. In regard to their current relationship status, 71 (6.4%) were married, 518 (47.2%) in a relationship, and 508 (46.3%) were single.

Regarding associations with age a small positive correlation with the MI-RSWB 12 scales GR (*r* = 0.10; *p* < 0.001) was observed, while all other scales correlations remained insignificant (*p* > 0.001). No scale showed significant sex differences (F = 6.61–F = 1.12; all *p* > 0.001). Detailed descriptions of several scale characteristics can be retrieved from Table 1.

Assessment of multivariate normality suggested non-normality of the data (multivariate kurtosis = 30.72, critical ratio = 27.75), hence results where bootstrap corrected.

Table 2 displays the correlation among the MI-RSWB scales. All subscales showed significant positive correlations with the RSWB-total score (*r* = 0.55–0.70; all *p* < 0.001). Furthermore, IWB was strongly related to HO (*r* = 0.77; *p* < 0.001) and FO (*r* = 0.80; *p* < 0.001), while GR (*r* = 0.28; *p* < 0.001) and CO (*r* = 0.21; *p* < 0.001) showed small correlations with IBW. This pattern was inversed for the TWB score: Large correlations were found with GR (*r* = 0.87; *p* < 0.001) and CO (*r* = 0.85; *p* < 0.001), while correlations with FO (*r* = 0.13; *p* < 0.001), and HO (*r* = 0.33; *p* < 0.001) were small.

**Table 2:** Correlations among variables.

**Variable**	**1**	**2**	**3**	**4**	**5**	**6**	**7**
1. General Religiosity	–						
2. Forgiveness	0.15^***^	–					
3. Hope	0.29^***^	0.24^***^	–				
4. Connectedness	0.49^***^	0.07	0.27^***^	–			
5. IWB	0.28^***^	0.80^***^	0.77^***^	0.21^***^	–		
6. TWB	0.87^***^	0.13^***^	0.33^***^	0.85^***^	0.29^***^	–	
7. RSWB	0.75^***^	0.55^***^	0.66^***^	0.70^***^	0.76^***^	0.84^***^	–

*N = 1,097*;

*** *p < 0.001; IWB, Immanent Well-Being; TWB, Transcendental Well-Being; RSWB, Religious/Spiritual Well-Being*.

### Confirmatory Factor Analyses of the MI-RSWB 12

As shown in Table 3 the results of the Confirmatory Factor Analysis (CFA) for the MI-RSWB 12 indicate that the single-factor model provided poor fit to the data, with none of the indices falling within the acceptable range [χ^2^/df = 46.61; RMSEA = 0.20 (90% CI: 0.19, 0.21); CFI = 0.54; NFI = 0.54; TLI = 0.44]. The single higher-order model showed generally acceptable fit [RMSEA = 0.05 (90% CI: 0.04, 0.06); CFI = 0.98; NFI = 0.97; TLI = 0.97], however exhibited a χ^2^/df > 3. In contrast, the four-factor [χ^2^/df = 2.75; RMSEA = 0.04 (90% CI: 0.03, 0.05); CFI = 0.98; NFI = 0.98; TLI = 0.98], two higher-order factors [χ^2^/df = 2.84; RMSEA = 0.04 (90% CI: 0.03, 0.05); CFI = 0.98; NFI = 0.97; TLI = 0.98] and the bifactor [χ^2^/df = 2.97; RMSEA = 0.04 (90% CI: 0.03, 0.05); CFI = 0.99; NFI = 0.98; TLI = 0.98] models showed overall good fit indices, with all indices falling within the acceptable range. Every model exhibited a Bollen-Stine bootstrap *p* = 0.000. With regard to the AIC values, the four-factor model demonstrated superiority compared to both the two higher-order factor model (ΔAIC = 5.44) and the bifactor model (ΔAIC = 4.8), while no significant differences are observed between the bifactor and the two higher-order factor models (ΔAIC = 0.64).

**Table 3:** Confirmatory factor analysis fit statistic for the MI-RSWB 12.

**Model**	**χ^2^(df)**	**χ^2^/df**	**RMSEA (90% CI)**	**CFI**	**NFI**	**TLI**	**AIC**
1. Single-factor	2517.12 (54)	46.61	0.204 (0.197–0.211)	0.54	0.54	0.44	2565.12
2. Four-factor	131.77 (48)	2.75	0.040 (0.032–0.048)	0.98	0.98	0.98	191.77
3. Single higher-order factor	175.58 (50)	3.51	0.048 (0.040–0.056)	0.98	0.97	0.97	231.58
4. Two higher-order factors	139.21 (49)	2.84	0.041 (0.033–0.049)	0.98	0.97	0.98	197.21
5. Bifactor	124.57 (42)	2.97	0.042 (0.034–0.051)	0.99	0.98	0.98	196.57

*N = 1,097; RMSEA, Root Mean Square Error of Approximation; CFI, Comparative Fit Index; NFI, Normed Fit Index; TLI, Tucker-Lewis Index; AIC, Akaike information criterion*.

**Figure 2:**
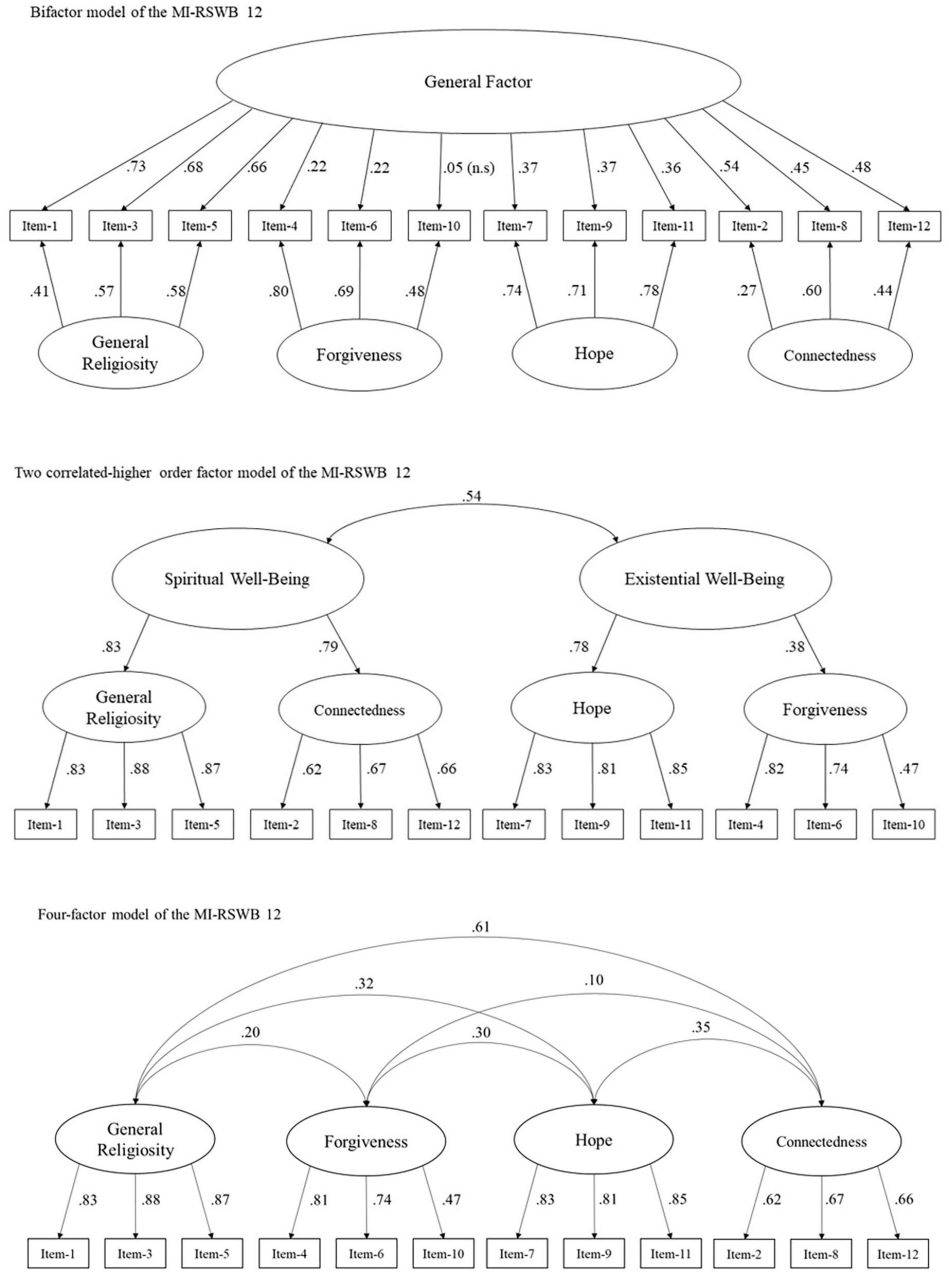
Inter-factor correlations and regression weights of models showing an acceptable model fit.

The results of the CFA for the four-factor, two higher-order factors and the bifactor models, estimated with a bootstrap ML (250 samples), are detailed in Figure 2. Regarding the bifactor model, all assigned regression weights were significant (*p* < 0.01), except for the association between item 10 and the general factor (*p* > 0.05). The estimated strengths of the significant associations between the general factor and the individual items ranged from β = 0.22 to 0.74. Furthermore, the items assigned to the GR factor showed stronger factor loadings onto the general factor (β = 0.66 to 0.74; all *p* < 0.01) than items assigned to the remaining dimensions of the MI-RSWB 12 (β = 0.22 to 0.54).

Concerning the two higher-order factor model, both higher-order factors were substantially correlated (*r* = 0.54; *p* < 0.01). Moreover, all assigned associations were significant (all *p* < 0.05–0.01). The higher-order factor TWB was substantially associated with both GR (β = 0.83) and CO (β = 0.79), while HO (β = 0.78), and FO (β = 0.38) were associated with IWB. Associations between the individual items and the dimensions of the MI-RSWB 12 ranged between β = 0.83 to 0.88 for GR, between β = 0.62 to 0.67 for CO, between β = 0.81 to 0.85 for HO, and between β = 0.47 to 0.82 for FO.

With regard to the four correlated factor model, the associations between the individual items and the MR-RSWB 12 dimensions remained unchanged. Correlations between the factors ranged from *r* = 0.10 (FO × CO; *p* < 0.05) to *r* = 0.61 (GR × CO; *p* < 0.01). All remaining correlations were significant (all *p* < 0.01).

### Invariance Analysis

As detailed in Table 4 the examination of the CFI difference between Model 1 and Model 0 suggested invariance of the factor loadings across the male and female participants (ΔCFI ≥ −0.01). However, the CFI difference between Model 2 and Model 1 indicates that there is no complete invariance of the intercepts across the two groups. Following the recommendation to free one item intercept at a time (Dimitrov, 2010), further analysis resulted in a modified model which was labeled Model 2P. After freeing the intercept for Item 5 a ΔCFI ≥ −0.01 was achieved. Therefore, with the exception of the intercept of one indicator (item 5) there are invariant factor loadings and intercepts across both groups. Model 3 was obtained from Model 2P by constraining the variances and covariances of the items' residuals. Based on the comparison of the CFI of Model 3 and Model 2P, item invariance of item uniqueness can be assumed (ΔCFI ≥ −0.01). Finally, Model 4 was obtained from Model 2P by imposing invariant factor variances and covariances. The difference regarding CFI between Model 4 and Model 2P remained ΔCFI ≥ −0.01, thus, indicating structural invariance with regard to both groups.

**Table 4:** Testing for invariance across gender.

**Model**	**χ^2^**	**df**	**Model Comparison**	**CFI**	ΔCFI^*^
M0	207.145	96		0.979	
M1	218.699	104	M1–M0	0.980	0.001
M2	310.536	113	M2–M1	0.964	−0.016
M2P	251.888	112	M2P–M1	0.974	−0.006
M3	282.606	124	M3–M2P	0.971	−0.003
M4	291.889	125	M4–M2P	0.969	−0.005

*CFI, Comparative Fit Index; M0, Baseline model (no invariance imposed); M1, Invariant factor loadings; M2, Invariant factor loadings and intercepts; M2P, invariant factor loadings and partially invariant intercepts (free intercept of Item 5); M3, invariant factor loadings, partially invariant intercepts, and invariant residual variances; M4, Invariant factor loadings, item intercepts, and factor variances/covariances*;

* Δ*CFI ≤ −0.1 signals lack of invariance targeted by the respective comparision of nested models*.

## Discussion

It was intended in this study to further contribute to the flourishing research concerning the spiritual dimension within the area of psychological well-being, whereby we applied the Multidimensional Inventory for Religious/Spiritual Well-Being (MI-RSWB) in its original version with 48 items in various research settings. Based on these positive experiences a short version of the scale was constructed, especially for the application in clinical assessment. Hereby the original number of items were reduced from 48 items to 12 items (MI-RSWB 48 vs. MI-RSWB 12), as well as the originally six sub-dimensions were limited to four (GR, CO, HO, FO). Therefore, both sub-dimensions from the original MI-RSWB 48 scale: “Hope Transcendent” (HT) as well as “Experiences of Sense and Meaning” (SM) had to be deleted (Unterrainer et al., 2014).

Based on the comparison of different structural equation models for the structural validity of MI-RSWB 12, a correlated four-factor model can be preferred. Further analysis of this model suggested structural invariance with regard to gender. Hence, it can be assumed that the four-factor model of the MI-RSWB 12 operates in the same way with regard to its underlying structure for both male and female subjects (Dimitrov, 2010). In accordance with the theoretical basic assumptions about the scale, it can be concluded that it makes sense to calculate all four dimensions of the scale (GR, CO, HI, FO). What is more, our estimated models indicate the validity of a latent bifactor structure consisting of a general RSWB factor and the four domain specific factors (GR, CO, HI, FO), as well as a latent structure consisting of the two higher order factors TWB and IWB and the four lower order factors (GR, CO, HI, FO). These results underline the high flexibility of the MI-RSWB 12 regarding its ability to measure different facets of well-being not limited to the transcendent but also within the immanent realm of perception. Following Maslow (Maslow, 1964) both aspects of well-being might be seen as related, at least to a certain extent. The results of this study which indicate a substantial correlation between TWB and IWB further underline this assumption.

Interestingly, the bifactor model—while better fitting than the single higher-order factor model—did not show superior model fit compared to the two correlated higher-order factor model or the correlated four-factor model. This is in contrast with a great proportion of the literature on bifactor models. Several authors suggest that bifactor models tend to generate better fit indices than higher-order models (see e.g., Murray and Johnson, 2013; Cucina and Byle, 2017). Some researchers even argue that the comparison between bifactor and hierarchical models might be substantially biased in favor of the bifactor model (Murray and Johnson, 2013). Hence, the results of the present study further emphasize the structural multidimensionality of the MI-RSWB 12. However, this finding might be particularly dependent on item-10, which showed no significant loading regarding the general factor of the bifactor model.

As is well-known from previous studies, the dimensions of well-being that relate to the transcendent area of perception (GR and CO) were always shown to be less strongly linked to various parameters of mental health (e.g., increased mood stability or more adequate coping strategies) compared to the parameters for Immanent Well-Being (HI and FO). In correspondence to this, according to Unterrainer (2010), and Unterrainer et al. (2012), it can be assumed that HI and FO together can be seen as a particularly strong predictor of the Sense of Coherence parameter within the Salutogenesis model of Antonovsky (1987), which in turn can be assumed as a strong indicator of more adequate stress coping.

In terms of neural correlates of MI-RSWB, a voxel-based morphometry study regarding the belief in the miracles of Lourdes (Schienle et al., 2020) reported substantial correlations between the MI-RSWB dimensions and specific areas in the brain. In detail, the region of interest (ROI) analyses showed negative associations between hippocampus volume and the total RSWB score as well as the HI sub dimension. Furthermore, GR was observed to be negatively correlated with amygdala volume. Although the long version of MI-RSWB was applied in this study, both dimensions (HI and GR), for which neural correlates were observed, can also be found in the short version of the MI-RSWB scale. However, it has to be noted, that there were also substantial neural correlates for the HT-dimension in the Schienle et al. (2020) study.

Restrictively, HT is omitted in the MI-RSWB 12 version. The reason for excluding the HT subscale for the MI-RSWB 12 is, that the HT dimension always showed to be responsible for a significant deterioration of the model fit of the MI-RSWB 48 (Unterrainer et al., 2010b). Therefore, the HT scale was retained for the long version of the scale for reasons of content, but was consequently eliminated for the MI-RSWB 12. As already mentioned, HT represents an inverse correlate of the fear of death and dying as well as the overcoming of threatening feelings such as existential fear. Traditionally, the overcoming of the fear of death and dying and its relation to psychological well-being is extensively addressed within Terror Management Theory (Greenberg et al., 1986; Burke et al., 2010; for an enhanced theoretical discussion see Becker, 1997). In correspondence to this, for instance, Aberer et al. (2018) could show that the HT dimension could be exclusively addressed by a specially developed spiritually oriented therapeutic intervention in different groups of patients with severe skin diseases. Accordingly, after the intervention the skin-patients exhibited an increased amount of HT (which mirrors inversely a decreased amount of anxiety of death and dying) in comparison to a control group. These exciting insights into clinical work definitely deserve to be examined in more detail by means of further research. Either way, this important finding could not have been gained by applying only the MI-RSWB short version.

Accordingly, the dimension of HT deserves to be further discussed regardless of the RSWB concept or in relation to it.

A similar problem occurs in terms of the “Experiences of Sense and Meaning” (SM) dimension, which was also removed within the MI-RSWB 12. It is not for nothing that experiences of sense and meaning were extensively discussed within the context of nootherapeutic approaches (Viktor Frank and his Existential/Logotherapy can probably be mentioned here as the most prominent representative; Frankl, 1963). As part of the RSWB concept, the SM sub-scale often showed the most unconvincing psychometric properties and was therefore removed. However, in terms of content, it still appears to highly relevant, especially for more psychotherapeutically oriented research questions.

### Limitations and Future Perspectives

A constraint of this study is the non-normality of the investigated data which might have affected the results of the CFA. Hence, Bollen-Stine bootstrap was employed to manage the effects of the violated multivariate normal assumption in terms of corrected *p*-values for the χ^2^ statistic, adjusted standard errors and confidence intervals for parameter estimates. In correspondence, the significant Bollen-Stine bootstrap might be seen as problematic regarding the global fit of all investigated models (Bollen and Stine, 1992). Consequently, this result suggests a restriction of the structural validity of the MI-RSWB.

So far, little has been done regarding the changeability of the RSWB dimensions over several measurement times. There is still a lot of work to be done here and a concise instrument seems to be preferable in many cases within a repeated measurement design. Based on the limited findings of previous research, it can be assumed that the RSWB dimensions can be changed in principle. However, these dimensions may need to be specifically addressed by means of a uniquely developed treatment protocol (e.g., Sollgruber et al., 2018).

In conclusion, the MI-RSWB 12 short scale proves to be a very reliable instrument, with an excellent structural validity. Therefore, the MI-RSWB 12 is an adequate alternative for the long form of the scale (MI-RSWB 48; Unterrainer et al., 2010b), especially regarding clinical applications in vulnerable groups, such as patients in psychiatric treatment, general medical inpatients or people in prison.

## Data Availability Statement

The data analyzed in this study is subject to the following licenses/restrictions: Data set can be retrieved from the corresponding author. Requests to access these datasets should be directed to HU, human.unterrainer@univie.ac.at.

## Ethics Statement

The studies involving human participants were reviewed and approved by Medical University of Graz, Austria. The patients/participants provided their written informed consent to participate in this study.

## Author Contributions

All statistical analysis were conducted by JF. JF wrote the first draft of the manuscript. HU read the manuscript and made some critical comments. JF and HU revised the whole manuscript together. Both authors contributed to the article and approved the submitted version.

## Conflict of Interest

The authors declare that the research was conducted in the absence of any commercial or financial relationships that could be construed as a potential conflict of interest.
