# Supplementary material for: “Test Your Spirituality in One Minute or Less” Structural Validity of the Multidimensional Inventory for Religious/Spiritual Well-Being Short Version (MI-RSWB 12)
Source: Front Psychol. 2021 Feb 2;12:597565. doi: 10.3389/fpsyg.2021.597565 (PMC7884348; doi:10.3389/fpsyg.2021.597565)
Supplement: Supplementary file 1 [file Data_Sheet_1.docx]

Appendix:

A1

Instruction and Items for the Multidimensional Inventory for Religious/Spiritual Well-Being (MI-RSWB 12) in English language together with a short manual.

**Instruction (English):**

Please keep the following in mind as you answer the questions: 1. You will find several questions concerning your religious/spiritual beliefs and you will often come across the term “God”. 2. Your answers will only be used for research purposes and you will remain completely anonymous. 3. This study was not sponsored by a religious group and data will not (in their entirety or in part) be passed on to such organizations. 4. If you feel uncomfortable with the term God, feel free to substitute it with a term you find suitable, such as “higher power” for example. 5. It is also possible to complete the questionnaire if you have agnostic or atheistic beliefs - the questionnaire takes such beliefs into consideration. Please respond quickly and try not to take too long deliberating upon any one question. It is also important to answer each and every question; failing to respond to all the questions means that it will not be possible to evaluate the questionnaire correctly.

Table A1

*Items and dimensions of the MI-RSWB (English version)*

| Nr. | Dim. | Item |
| --- | --- | --- |
| 1 | GR | My faith gives me a feeling of security. |
| 3 | GR | It is possible for me to find contentment in intimate conversations with God. |
| 5 | GR | I will be able to overcome all problems with God's help. |
| 4 | FO | There are people whom I hate.* |
| 6 | FO | There are people whom I will never be able to forgive.* |
| 10 | FO | If somebody hurts me, I usually try to get revenge.* |
| 7 | HO | I think my life is moving in the right direction. |
| 9 | HO | I think that I will have more positive than negative experiences in the future. |
| 11 | HO | I think that I will live my life in the future just as I envisage it. |
| 2 | CO | I have experienced the feeling of being absorbed into something greater. |
| 8 | CO | There are people with whom I feel a supernatural connection. |
| 12 | CO | I have experienced things which radiate a special kind of energy. |

*Note*. Nr.= Item number in the Questionnaire; Dimensions: GR = General Religiosity; FO = Forgiveness; HO = Hope; CO = Connectedness. Likert scale: 1: totally disagree – 6: totally agree. Items marked with * have be to coded inverse.

A2

Instruction and Items for the Multidimensional Inventory for Religious/Spiritual Well-Being (MI-RSWB 12) in German language together with a short manual.

**Instruction (German):**

Bevor Sie an die Beantwortung der Fragen gehen, bitte ich Sie folgende Dinge zu beachten: Sie werden zum großen Teil Fragen bezüglich Ihres religiösen Glaubens finden. Auch werden Sie bei der Bearbeitung der einzelnen Fragen öfters auf den Begriff „Gott“ stoßen. Dazu ist wichtig anzumerken:   Die hier gemachten Angaben dienen ausschließlich der wissenschaftlichen Forschung und werden vollkommen anonym ausgewertet. Diese Untersuchung wurde von keiner religiösen Vereinigung oder Gemeinschaft gefördert, noch werden Daten an eine solche weitergegeben. Der Begriff „Gott“ kann, muss aber nicht im christlich– konfessionellen Sinne verstanden werden. Falls Sie den Begriff „Gott“ als unpassend empfinden, können Sie diesen gedanklich durch eine Formulierung wie „höhere Macht“ ersetzen. Die Beantwortung des Fragebogens ist trotz der völligen Ablehnung eines religiösen Glaubens möglich. Diese Haltung wird im Fragebogen berücksichtigt. Bitte beantworten Sie die Fragen spontan und halten Sie sich gedanklich nicht zu lange bei einer Frage auf. Weiters bitte ich Sie jede Frage zu beantworten, da nur so eine korrekte Auswertung möglich ist.

*Table A2: Items and dimensions of the MI-RSWB 12 (German version)*

| Nr. | Dim. | Item |
| --- | --- | --- |
| 1 | GR | Mein Glaube gibt mir ein Gefühl von Sicherheit. |
| 3 | GR | Es ist für mich möglich, Zufriedenheit im vertraulichen Gespräch mit Gott zu finden. |
| 5 | GR | Ich werde mit Gottes Hilfe alle Probleme bewältigen können. |
| 4 | FO | Es gibt Menschen, die ich hasse.* |
| 6 | FO | Es gibt Menschen, denen ich niemals verzeihen werde können.* |
| 10 | FO | Wenn mich jemand verletzt hat, versuche ich in der Regel es ihm heimzuzahlen.* |
| 7 | HO | Ich glaube, mein Leben entwickelt sich in die richtige Richtung. |
| 9 | HO | Ich glaube, dass ich in Zukunft mehr positive Erlebnisse haben werde als negative. |
| 11 | HO | Ich glaube, dass ich in Zukunft so leben werde, wie ich mir das vorstelle. |
| 2 | CO | Ich habe eine Erfahrung gemacht, in der meine Person in etwas Größerem aufzugehen schien. |
| 8 | CO | Es gibt Menschen, zu denen ich eine übersinnliche Verbindung verspüre. |
| 12 | CO | Ich habe erfahren, dass es Gegenstände gibt, von denen eine ganz besondere Kraft ausströmt. |

*Note*. Nr.= Item number in the Questionnaire; Dimensions: GR = General Religiosity; FO = Forgiveness; HO = Hope; CO = Connectedness. Likert scale: 1: totally disagree – 6: totally agree. Items marked with * have be to coded inverse.
